# Genetic Analysis of Italian Local Apple Genotypes from the Abruzzo Region

**DOI:** 10.3390/genes17070771

**Published:** 2026-06-30

**Authors:** Sabrina Lucchetti, Emanuele Masillo, Valentina Melini, Francesca Melini, Francisco Javier Comendador Azcarraga, Roberto Ambra

**Affiliations:** 1 Council for Agricultural Research and Economics (CREA), Research Centre for Food and Nutrition, 00178 Rome, Italy; sabrina.lucchetti@crea.gov.it (S.L.); valentina.melini@crea.gov.it (V.M.); francesca.melini@crea.gov.it (F.M.); fjavier.comendador@crea.gov.it (F.J.C.A.); 2 MEMOTEF Department, Sapienza University of Rome, 00185 Rome, Italy; emanuele.masillo@uniroma1.it

**Keywords:** *Malus domestica* Borkh., apple, local varieties, Cerina, Gelata, Limoncella, Renetta, Rosa, Zitella, Golden Delicious, SSR markers, agrobiodiversity conservation, molecular characterization, varietal traceability, genetic diversity, genetic structure

## Abstract

**Objectives**: Within the framework of the European Union’s Submeasure 10.2 for the conservation and sustainable use and development of genetic resources in agriculture, this study aimed to genetically characterize specific apple (*Malus domestica* Borkh.) varieties from the Abruzzo region (Italy) using SSR (Simple Sequence Repeat) markers. **Methods**: A total of 67 samples, including 10 reference accessions from a national germplasm collection, were analyzed to assess genetic diversity and varietal traceability. Nine highly polymorphic SSR markers were selected based on a literature review and applied to DNA extracted from leaf tissues. **Results**: Molecular analyses, including PCR amplification and capillary electrophoresis, revealed seven genetic groups with identical profiles and 15 individuals with unique genetic fingerprints, representing approximately 22% of the dataset. Statistical analyses using R Studio (PAST and LEA) confirmed high genetic diversity (mean PIC = 0.81; mean He = 0.83) and identified two major genetic clusters, with evidence of admixed ancestry in some accessions. **Conclusions**: These findings highlight discrepancies between declared and actual varietal identities, underscore the value of genetic traceability for biodiversity conservation, and provide a robust framework for the protection and valorization of local apple germplasms.

## 1. Introduction

Biodiversity encompasses the variety of life forms present on Earth and plays a crucial role in both environmental sustainability and human nutrition. At the global level, the diversity of domesticated plant varieties and animal breeds is steadily declining. This erosion of biodiversity, particularly in its genetic component, due to habitat alteration, climate change, overuse of resources, and human socioeconomic pressure, represents a significant concern for food security, as it weakens the capacity of agricultural systems to respond effectively to pressures such as pests, diseases, and climate change [1]. Agricultural systems that are rich in biodiversity are more resilient to environmental stressors and provide a broader genetic base for the selection of more resistant crops varieties and livestock breeds. This contributes significantly to addressing challenges related to food security, in line with the objectives of the United Nations 2030 Agenda for Sustainable Development. Moreover, biodiversity is deeply intertwined with cultural identity, reflected in local culinary traditions and traditional farming practices, and serves as a valuable source of scientific knowledge and innovation [2].

Genetic diversity underpins biodiversity, as genetic variation enables populations to adapt and ensures better survival under environmental change. Assessing genetic diversity, that is, measuring how much genetic variation exists within or between populations, can be achieved by several approaches such as Simple Sequence Repeats (SSRs), SNPs (Single Nucleotide Polymorphisms) and DNA sequencing.

Among these, SSRs—short tandemly repeated DNA sequences—are widely distributed across genomes, highly polymorphic, co-dominant, easy to use, and yield highly reproducible results, making them suitable for applications ranging from forensic analysis and varietal classification to population genetics, biodiversity protection, and the characterization of highly processed food matrices. The analysis of SSR polymorphisms enables the construction of genetic fingerprints, or molecular identity cards, allowing the unique identification of individuals, varieties, and matrices—even after undergoing transformation processes—and the reconstruction of genetic relationships through phylogenetic analyses. As other molecular markers, SSRs allow for overcoming the main limitations of traditional traceability methods based on morphological or chemical descriptors, which are often strongly influenced by environmental conditions and post-harvest processing, thus reducing their reliability and reproducibility [3]. In this study, genetic diversity was investigated using an integrated approach that combines molecular markers and statistical analyses, ensuring a robust assessment of variability and relatedness.

Despite the growing application of molecular markers in the characterization of plant genetic resources [4], many local and traditional germplasm collections remain insufficiently explored. In Italy, numerous local apple varieties have been historically conserved through traditional agricultural practices, thanks mainly to individual initiatives, often in marginal environments. These autochthonous genotypes represent a valuable reservoir of genetic diversity, potentially harboring unique alleles associated with adaptation to local conditions and resistance to biotic and abiotic stresses. However, to date, comprehensive molecular data on these varieties are largely lacking, and their genetic relationships and diversity structure remain poorly understood. This gap of knowledge limits both their effective conservation and their potential exploitation in breeding and traceability systems. Therefore, the present study aims to genetically characterize a wide collection of local apple varieties from the Abruzzo region using SSR markers, to assess their genetic diversity, infer relationships among genotypes, and contribute to the valorization and preservation of this underexplored germplasm.

## 2. Materials and Methods

### 2.1. Plant Material

Plant samples were collected from various orchards located in the Abruzzo region of Italy, across the provinces of L’Aquila, Teramo, and Pescara. According to the information provided by the producers, the samples were derived from six local apple varieties, as well as from the Golden Delicious cultivar. Specifically, the dataset included Cerina (*n* = 2), Gelata (*n* = 12), Limoncella (*n* = 18), Renetta (*n* = 14), Rosa (*n* = 5), Zitella (*n* = 5), and Golden Delicious (*n* = 1) (Table 1). Regarding the Cerina samples, the producer indicated that they corresponded to Gelata specimens, in agreement with the synonymy reported in the literature [5].

In order to compare the genetic identity of the different accessions, we included in the study 10 reference samples provided by CREA-OFA (Research Centre for Olive, Fruit and Citrus Crops) located in Rome (Italy), which maintains a germplasm collection of ancient and native varieties. Specifically, the reference set comprised one sample each of Cerina, Limoncella, Renetta Rossa, Renetta Verde, Renetta Ruggine, Rosa, Zitella, Golden Delicious Chic, Golden Delicious Emla, and Golden Delicious Holcome. In the paper, these reference samples from CREA-OFA are identified using Sample IDs that exactly match the variety names.

Overall, the number of samples analyzed was 67, including the 10 from the CREA-OFA catalogue field. From each plant, five to ten leaves were collected, and samples were labeled, transported to CREA-AN, and stored in a freezer at −80 °C to preserve DNA integrity.

### 2.2. DNA Extraction

Total genomic DNA was extracted from leaf samples ground directly into 1.5 mL tubes of the Tissue Grinding Tool (cat. E0359, EURx, Gdansk, Poland), using pestles and grinding beads provided in the kit. Extraction solutions were derived from the Plant Genomic DNA Mini Kit (cat. GP100, New Taipei City, Taiwan), employed following a modified protocol. Approximately 50 mg of sliced leaves was disrupted by grinding for 2 min at RT in 200 µL of GP1 Buffer and 5 µL of RNase A. An additional 200 µL of GP1 Buffer weas then added, and the homogenate was incubated at 60 °C for 15 min, inverting the tube every 5 min. Subsequently, 100 µL of cold GP2 Buffer was added. The lysate was vortexed briefly, incubated on ice for 5 min and centrifuged at maximum speed for 5 min. The supernatant was further clarified by centrifugation through filter columns for 1 min at 3800 rpm. DNA binding, washing and elution were performed according to the Plant Genomic DNA Mini Kit protocol, including a wash step with absolute ethanol and using 100 µL pre-heated elution buffer. Quality and quantity of DNA samples were checked using a NanoDrop ND-1000 spectrophotometer (NanoDrop Technologies Inc., Montchanin, DE, USA).

### 2.3. Selection of SSR Markers

The selection of SSR markers for genotyping apple samples was performed after a review of the existing literature on the genetic characterization of Italian apple accessions [5,6,7,8,9,10]. From each of the six studies identified, the most polymorphic SSRs, i.e., those producing the highest number of alleles within the analyzed populations, were selected. Among the reviewed works, the studies by Liang et al. [9] and Marconi et al. [5] were the most extensive, genotyping 275 and 175 apple accessions, respectively. The dataset in the study by Liang et al. included all the five local varieties analyzed in this study, whereas the study by Marconi et al. [5] covered three of them (i.e., Rosa, Limoncella, and Gelata). Guarino et al.’s work analyzed 27 unique accessions, including two of the local varieties (Zitella and Limoncella) [8]. The studies by Cavanna et al. [7], Testolin et al. [10] and Alessandri et al. [6] examined 44, 78, and 47 accessions, respectively, but included only one local target variety (Renetta in Cavanna et al.; Rosa in the other two studies). Given these differences, an arbitration criterion based on proportionality was applied: from each study, the number of SSRs chosen corresponded to the number of our varieties represented, with the exception of Liang’s study, from which only the four most polymorphic examples were selected. This yielded a set of nine unique SSRs.

Primers’ efficiency and the correct size of the amplicons were initially checked, resolving PCR products on precast 2% E-Gel™ Agarose Gels run on an Invitrogen™ E-Gel™ Power Snap Plus Electrophoresis System using the E-Gel™ 50 bp DNA Ladder (Thermo Fisher Scientific, Waltham, MA, USA) as molecular weight standard. PCR reactions were run in a SimpliAmp Thermal Cycler (Thermo Fisher Scientific, Waltham, MA, USA) in a total volume of 25 µL containing 100 ng of DNA, 2.5 µM of each primer (Bio-Fab Research, Roma, Italy) and 1x onHybrid PCR Master Mix (EurX, Gdansk, Polska). DNA amplification conditions comprised an initial denaturation step of 95 °C for 10 min, 35 cycles of denaturation at 95 °C for 30 s, appropriate annealing temperature for 30 s and extension at 72 °C for 30 s. A final extension cycle was performed at 72 °C for 7 min.

### 2.4. SSR Fingerprinting

PCRs for SSR fingerprinting were performed as mentioned above but using modified primers with either Cy3, 6-Fam or Hex fluorophores attached at the 5′-end of the respective forward sample. For each sample, fragments sizes were extrapolated by capillary electrophoresis separation, i.e., three post-PCR multiplexed (MP) pools of three individual PCR assays appropriately combined with the three Cy3, 6-Fam and Hex fluorophores, in order to limit overlapping among the expected dimensions of the amplicons (Table 2).

Pooled PCRs were run on an ABI 3730XL Genetic Analyzer (at Bio-Fab Research, Roma, Italy) under the following conditions: 10 s injection time, 1.6 kV injection current, 2100 s run time, 15 kV run current, 50 cm capillary length, POP7 Polymer, Dye Set G5 filter. A GeneScan™ 500 LIZ™ device was used as the size standard (Thermo Fisher Scientific, Waltham, MA, USA), and chromatograms of SSR markers were analyzed using Microsatellite Analysis Software (MSA) (Software version 1.2) genotyping module available online at the Thermo Fisher Cloud (https://apps.thermofisher.com/editor-web/#/app/app-microsatellites-web) (accessed on 30 January 2026).

### 2.5. Data Analysis

SSR markers from the 67 apple accessions were analyzed using RStudio (release 4.5.1) to assess a quantitative evaluation of the genetic diversity present and to provide insights into the underlying evolutionary and selective processes. For each SSR locus, the following parameters were calculated: the total number of alleles (Na), the effective number of alleles (Ne), the percentage of rare alleles (Ra), the observed heterozygosity (Ho), the expected heterozygosity (He), the inbreeding coefficient (F), and the polymorphic information content (PIC) [11]. Ho reflects the proportion of heterozygous individuals, while He indicates the probability of allele diversity under Hardy–Weinberg equilibrium. F measures deviations from this equilibrium, with negative values indicating heterozygote excess and positive values suggesting deficiency.

The *He*, *Ho* and *PIC* value for each marker was computed according to the following formulas, where $p_{i}$ is the frequency of the i-th allele:

$$Ho=\frac{N_{heterozygotes}}{N}$$

$$He=1-\sum_{i=1}^{n}p_{i}^{2}$$

$$PIC=1-\sum_{i=1}^{n}p_{i}^{2}-{\left(\sum_{i=1}^{n}p_{i}^{2}\right)}^{2}+\sum_{i=1}^{n}p_{i}^{4}$$

**Figure 1:**
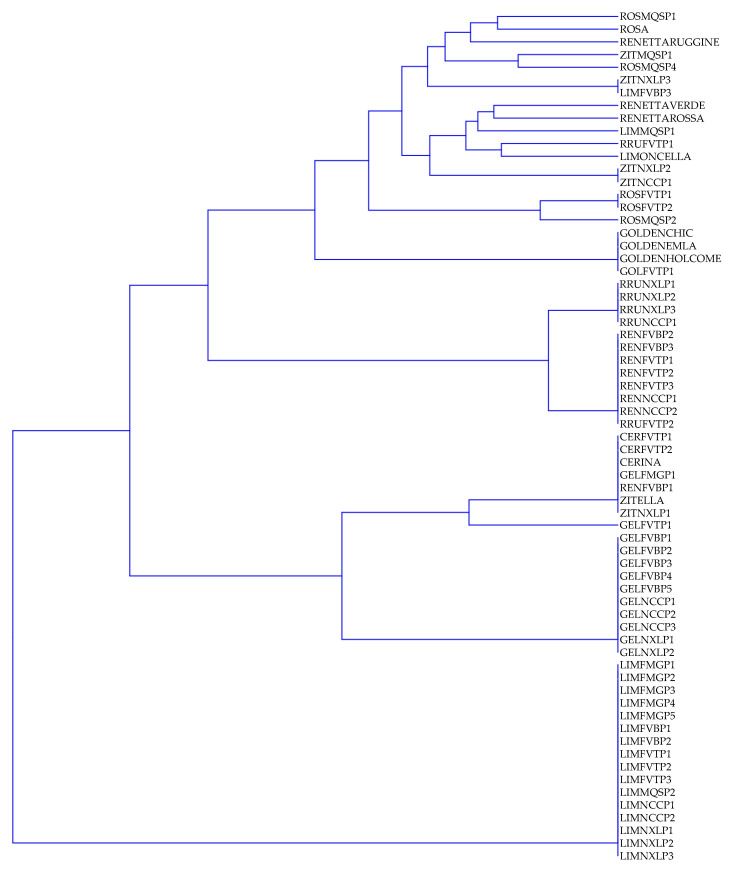
Dendrogram of hierarchical clustering of the 67 apple accessions, according to Ward’s method.

For cluster analysis, a binary presence/absence matrix was generated for each individual to encode the occurrence of alleles, comprising accessions with potentially mixed polyploid characteristics. Clustering was performed following Ward’s hierarchical method [12], using the R software and a Euclidean distance matrix. The resulting dendrogram (see Figure 1) identified groups of individuals with an identical genetic profile (zero distance), which were removed in order to avoid bias in the analysis. After duplicate removal, remaining individuals were used again as binary data for a cluster analysis based on Ward’s method. The optimal number of genetic clusters (K) was estimated using the LEA package for R [13], which implements admixture models based on a matrix factorization approach to infer population genetic structure. This framework is conceptually analogous to the Bayesian models used in STRUCTURE [14], but allows for a detailed characterization of each individual’s genetic composition by detecting admixture signals, i.e., partial membership of individuals across multiple genetic clusters.

The model was run 10 times independently for each value of K between 1 and 7, and the optimal K number was determined using the cross-entropy criterion, which evaluates the model’s predictive ability on a fraction of artificially masked genetic data (masked genotypes) [15]. Specifically, a subset of genotypes is temporarily removed from the dataset and then predicted by the model using estimated allele frequencies and individual ancestry proportions. Cross-entropy measures the mismatch between observed and predicted genotypes, providing an index of model fit, with lower values indicating a better ability to capture of the underlying genetic structure [16]. The analysis was repeated 100 times, recording the value of K associated with the minimum cross-entropy for each run, and the optimal number of clusters was then defined as the K value most frequently observed across all replicates. According to this criterion, the optimal number of genetic clusters was K = 2.

To evaluate the robustness of the clustering results, an additional clustering analysis was performed using Bruvo’s distance [17] and the UPGMA algorithm [18]. Bruvo’s distance provides a biologically meaningful estimate of genetic distance, especially for SSR data, accounting for differences in allele length through a stepwise mutation model. The UPGMA algorithm uses the resulting genetic distance matrix to progressively cluster individuals, allowing the identification of genetically related groups and facilitating comparison with the clustering pattern obtained from the binary presence/absence data. The dendrograms obtained using the two clustering methods on the same dataset identified the same set of individuals, with zero genetic distance, and yielded consistent results after repeating both analyses following duplicate removal, including the number of genetic clusters. This agreement indicates that the conversion of allelic data into a binary format did not lead to a significant loss of genetic information. Therefore, only the clustering analysis based on the binary presence/absence matrix is reported here.

## 3. Results

### 3.1. SSR Polymorphism and Genetic Diversity

Table 3 reports the main genetic diversity parameters calculated using the nine SSR loci analyzed in the *M. domestica* population. Overall, the markers displayed a good level of polymorphism and diversity. The number of alleles per locus (Na) ranged from 10 (CH02d08, CH01g12) to 15 (CH03d12, CH01a09), with an average of 12.22, indicating a high allelic richness for the selected markers. The effective number of alleles (Ne), which takes into account the allele frequencies, varies from 5.61 (CH04c07) to 7.45 (CH01a09), with an average of 6.37, equal to half of the total number of alleles, suggesting a relatively balanced distribution of alleles within the population.

The percentage of rare alleles (alleles whose frequencies are <0.01) ranged between 0% (CH01g12) to 36% (Hi05e07), with an average value of 17.96%. The occurrence of rare alleles reflects hidden genetic variability and suggests that some loci (such as Hi05e07) might have captured exclusive or private diversity present in specific accessions. Table 4 lists rare alleles (frequency < 0.01) for each locus, together with the corresponding accessions in which they were detected. The information content of the markers, expressed by the PIC value, was generally high (mean = 0.82), with maximum values observed in CH01a09 (0.853), CH01f02 (0.837) and CH03d12 (0.835), indicating the strong discriminatory power of these markers. Observed heterozygosity (Ho) ranged from 0.66 (Hi05e07) to 1 (CH04c07), with a mean value of 0.85, whereas expected heterozygosity (He) remained consistently high, averaging 0.84. These values indicate high genetic diversity at the population level. Several loci exhibited Ho values higher than those of He (CH03d07, CH04c07, CH01a09, CH01g12), suggesting an excess of heterozygotes, while others showed a deficit (e.g., Hi05e07), potentially attributable to inbreeding, genetic drift, or selective pressures. The inbreeding coefficient (F) ranged from −0.217 (CH04c07) to 0.215 (Hi05e07). Negative values (e.g., CH04c07, CH01g12) indicate an excess of heterozygotes with respect to Hardy–Weinberg equilibrium, while positive values (e.g., Hi05e07, CH01f02) point to possible inbreeding or population structure. PIC values ranged from 0.798 (CH04c07) to 0.853 (CH01a09), with a mean of 0.82. These results confirm that the selected markers are highly informative (PIC > 0.5), suitable for genetic structure analysis, varietal fingerprinting, and association studies. Overall, the results highlight a good balance between observed genetic variability and the discriminatory power of the markers. The combination of high Na, He, and PIC values suggests that the set of loci used is highly informative and suitable for analyzing the genetic structure of the population.

### 3.2. Accession Identification

Based on the producers’ indications, six varietal groups were expected: Golden, Rosa, Limoncella, Gelata/Cerina, Renetta, and Zitella. Genotyping only partially confirmed these initial assignments. In fact, using a 100% identity criterion, the accessions were ultimately divided into nine groups of individuals sharing the genetic profile (Table 5).

The largest group was LIM, which included only accessions originally attributed by producers to Limoncella (16 out of 18). Unexpectedly, the LIMONCELLA CREA-OFA reference fell outside this group. This accession showed no allele sharing with any other samples included in the study (see Table S1), while the two excluded accessions (LIMMQSP1 and LIMFVBP3) shared only the CH05e03 allele, even though LIMFVBP3 was identical to one accession originally attributed to Zitella (ZITNXLP3), resulting in their grouping within the combined LIM/ZIT group.

With respect to the CREA-OFA ZITELLA reference, it was found to be genetically identical to the CERINA reference, thereby disproving the expected Gelata/Cerina homonymy and supporting the creation of a combined ZIT/CER group. This group included both accessions originally attributed by producers to Cerina (CERFVTP1 and CERFVTP2) but only one to Zitella (ZITNXLP1). Additionally, one Renetta (RENFVBP1) and one Gelata (GELFMGP1) accession clustered within this group. Two other Zitella accessions (ZITNCCP1 and ZITNXLP2) were found to be identical and were therefore grouped together within the ZIT group. The remaining Zitella sample (ZITMQSP1) was genetically distinct, as it shared no more than one locus (CH05e03) with the other Zitella samples (Table S1).

With the exception of two accessions (the previously mentioned GELFMGP1 and GELFVTP1), all the remaining accessions classified by producers as Gelata were genetically identical and shared three out of nine loci analyzed within the ZIT/CER group. This group, designated as GEL, represented the second largest group (*n* = 10), containing exclusively accessions initially labeled as Gelata. However, their definitive attribution to the Gelata variety could not be confirmed due to the lack of a CREA-OFA reference accession. Regarding the two Gelata samples excluded from the GEL group, GELFMGP1 shared three SSR loci with the GEL profile, whereas GELFVTP1 shared only two loci with the profile (Table S1). Notably, GELFMGP1 fell within the previously described ZIT/CER group, while GELFVTP1 did not group with any other accession.

Most of accessions identified by producers as Renetta (*n* = 12 out of 14) were structured into two subgroups of genetically identical accessions, differing at only one SSR locus (Hi05e07). The largest subgroup, REN1, included RENFVBP2, RENFVBP3, RENFVTP1, RENFVTP2, RENFVTP3, RENNCCP1, RENNCCP2 and RRUFVTP2. Subgroup REN2 comprised RRUNXLP1, RRUNXLP2, RRUNXLP3 and RRUNCCP1. Among the two accessions falling outside the REN group were the previously mentioned RENFVBP1 (grouping within CER/ZIT) and RRUFVTP1. Unexpectedly, the three CREA-OFA Renetta reference accessions (RENETTAROSSA, RENETTARUGGINE and RENETTAVERDE) were genetically distinct from both the REN1 and REN2 profiles, sharing no more than one SSR locus (Table S1).

The ROS group comprised only two Rosa accessions (ROSFVTP1 and ROSFVTP2) out of the five originally indicated by the producers. The remaining accessions (ROSMQSP1, ROSMQSP2 and ROSMQSP4), as well as the CREA-OFA ROSA reference accession, were all genetically distinct, sharing no more than four out of the nine SSR loci analyzed, as observed for ROSMQSP2 (Table S1).

Group GOL included the accession GOLFVTP1 and the three CREA-OFA Golden Delicious reference accessions (GOLDENCHIC, GOLDENEMLA and GOLDENHOLCOME), which were genetically identical across all the SSR loci tested.

Overall, 12 accessions showed a discrepancy with respect to producers’ information. Among these, six grouped differently (CERFVTP1, CERFVTP2, GELFMGP1, RENFVBP1, LIMFVBP3 and ZITNXLP3), and six remained ungrouped (GELFVTP1, LIMMQSP1, ROSMQSP1, ROSMQSP2 and ROSMQSP4, ZITMQSP1). With respect to the CREA-OFA reference accessions, five remained ungrouped, as they resulted in genetically distinct accessions (RENETTAROSSA, RENETTARUGGINE, RENETTAVERDE, LIMONCELLA and ROSA).

### 3.3. Genetic Structure Analysis

The first hierarchical clustering analysis, performed using Ward’s method and Euclidean distance, revealed the presence of several individuals grouped at zero distance (Figure 1), confirming the existence of accessions sharing an identical genetic profile (Table 3).

**Figure 2:**
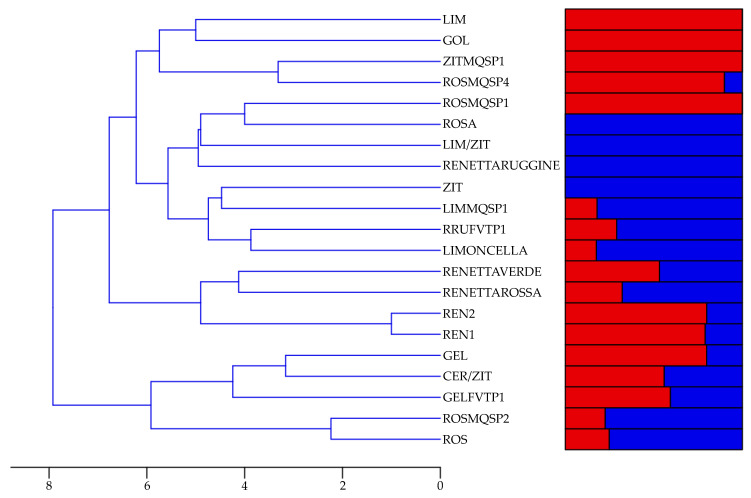
Dendrogram of hierarchical clustering (Ward’s method) of the 21 apple accessions (left) and assignment of genetic groups by LEA for K = 2 (right).

After removing 46 duplicated genotypes, the remaining 21 genotypes were reanalyzed using hierarchical clustering based on Ward’s method. In parallel, the genetic structure of the population was assessed using the LEA package. The results obtained (Figure 2) were aligned and compared to evaluate the consistency between the two analytical approaches.

**Figure 3:**
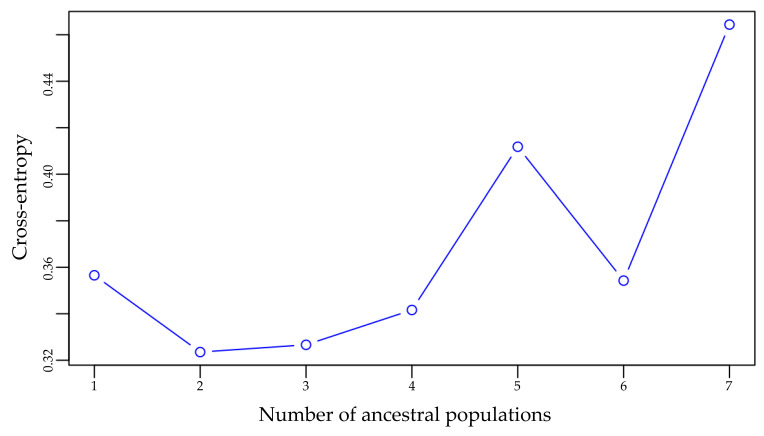
Estimation of the optimal number of clusters (K) via cross-entropy.

The hierarchical clustering analysis identified two main genetic groups within the dataset (Figure 3), providing a clear overview of the population structure.

The dendrogram generated using Ward’s method and Euclidean distance reveals two principal macro-clusters, consistent with the subdivision suggested by the genetic structure analysis performed with LEA. The additional branching within each macro-cluster highlights a non-negligible degree of internal variability among the accessions.

The LEA bar plot for K = 2 illustrates the probabilistic genetic composition of each individual. Most accessions display a clear assignment to one of the two genetic groups, as indicated by the predominance of a single color. However, a subset of individuals exhibits a more heterogeneous profile, reflecting admixture processes and suggesting either shared ancestral components or potential hybridization events.

LEA analysis therefore reveals an underlying genetic structure that may not be fully captured by hierarchical clustering alone. Nonetheless, the two approaches are largely consistent in identifying two principal groups, reinforcing the evidence for a well-defined genetic structure within the analyzed population. The differences observed can be attributed to the distinct methodological frameworks of the two approaches: hierarchical clustering relies on global genetic distances, whereas LEA employs a probabilistic model of ancestral group membership, enabling the identification of varying levels of genetic admixture.

## 4. Discussion

In the context of apple growing, the conservation of local varieties is not only a scientific priority but also a cornerstone of regional cultural identity and culinary traditions. Our study, even if conducted on a relatively small and unevenly represented sample of local apple varieties from the Abruzzo Italian region, highlights the importance of genetic characterization for biodiversity conservation and food quality protection.

The use of SSR molecular markers has proven to be a robust and highly informative approach, overcoming the limitations of traditional methods based on morphological descriptors, which are often influenced by environmental factors. From a methodological perspective, a previous study from Urrestarazu et al. [19] applied a wider common set of 16 SSR markers recommended by the European Cooperative program on Plant Genetic Resources (ECPGR). Our study utilized a specific set of nine SSR markers, selected following a review of available studies [5,6,7,8,9,10] focusing specifically on local Italian apple accessions (Rosa, Limoncella, Gelata, Zitella and Renetta) and applying a criterion of proportionality based on the representation of the target varieties (see Section 2.3. Selection of SSR Markers), to maximize discriminatory power and capture the specific variability of the national germplasm. Nonetheless, there is a partial overlap between the two sets, with four markers being shared between our study and that of Urrestarazu et al.: CH03d07, CH02d08, CH01f02, and CH04c07 [7,10,19].

The genetic diversity observed in the Abruzzo accessions (Na = 12.22; He = 0.84; PIC = 0.82) is high and consistent with previous SSR studies conducted on Mediterranean and European apple germplasms. Abruzzo varieties exhibit higher or comparable allelic richness than that of many regional and national collections. Within Italy, their diversity (Na = 12.22) exceeds that reported for Piedmont (10.9) [7] and the Rosa Romana accessions from the Apennines (9) [6] and is nearly identical to that of Friuli Venezia Giulia (12.3) [10]. Higher values are reported only for large “core” collections, such as those of the University of Bologna (16.87) [9] and the Edmund Mach Foundation (20) [20]. At the European level, the Abruzzo accessions show greater diversity than those from Lithuania (11.6) [21], Portugal (11.5) [22], and Sweden/Finland (8.78–9.78) [23]. Within the Mediterranean basin, they are comparable to those of Bosnia and Herzegovina (9.7) [24] and markedly higher than Moroccan varieties (1.69–5.23) [25], while remaining lower than only the Anatolian (Turkey) accessions (15.87) [26]. The high number of alleles per locus, together with the high content of polymorphic information (average PIC of 0.82), confirms that the selected markers possess strong discriminatory power, ideal for constructing “molecular identity cards” of local genotypes. The heterozygosity observed in Abruzzo (He = 0.84) is among the highest reported and even exceeds the continental average. It is higher than or comparable to values reported for pan-European germplasms (0.83 based on 1859 genotypes) [19], including those of Central Italy (0.81) [5], Spain (0.81) [27], Bosnia and Herzegovina (0.80) [24], and Sweden/Finland (0.72) [23]. In direct comparison, it aligns with the highest levels observed in the Madrid region (0.83–0.86) [28] and in Portugal (0.74–0.91) [22]. This finding indicates that the Abruzzo local varieties have not undergone significant genetic erosion and retain a high level of evolutionary plasticity, typical of Southern European gene pools. Moreover, the observation of an excess of heterozygotes at several loci suggests a dynamic population structure, although some positive inbreeding coefficient (F) values at certain markers indicate possible genetic drift or selective pressures in specific accessions. Regarding population structure, the identified K = 2 clusters in Abruzzo align with findings in Portugal [22] and larger Italian core collections [9], while being part of the broader Southern European (K1) gene pool identified in pan-European studies [19,27]. This group is characterized by a high number of private alleles—14 identified for Italy alone—suggesting specific long-term adaptation to the Mediterranean climate [19]. These comparisons place the Abruzzo germplasm as a critical regional component of the highly diverse European apple’s genetic heritage. Nevertheless, the uneven sample size across varieties (e.g., Cerina and Golden Delicious) limits the strength of inferences regarding intra-varietal diversity and population structure, and these findings should therefore be considered preliminary.

A central aspect concerns the known issue of trueness-to-type, or varietal authenticity. Historically, the exchange of plant material has led to numerous cases of synonymy (different names for the same genotype) and homonymy (same name for different genotypes). Our molecular analysis disproved several producer indications: samples attributed to the Zitella variety were found to be genetically identical to Cerina (forming the ZIT/CER group), refuting the synonymy with Gelata often cited in oral tradition. Guarino et al. [8] encountered similar results in Campania, where 27 out of 56 accessions were identified as synonyms, illustrating how names are often reassigned as material moves between orchards. Moreover, while the local Limoncella accessions formed a consistent genetic group, they did not match the official CREA-OFA reference, suggesting either a historical misnaming or a clonal divergence, as identified in Rosa Romana by Alessandri et al. [6]. For the Renetta variety, genetics also revealed a complex situation, with samples divided into two distinct subgroups (REN1 and REN2) that differ by only one locus but remain genetically distant from the official references of Renetta Rossa, Ruggine, or Verde. In contrast, the Golden Delicious accessions showed complete identity with the reference samples, confirming the genetic stability of this commercial cultivar. In total, six accessions of the Abruzzo population displayed a unique, non-shared genetic profile, corresponding to approximately 11% of the entire sample. Their genetic distinctiveness may reflect divergent origins, spontaneous mutations, or the presence of rare local varieties that—consistent with the results—are not aligned with OFA reference controls. Regardless of their origin, these genotypes represent a valuable reservoir of biodiversity, contributing to the enrichment of the overall genetic landscape. Overall, the results validate much of the traditional classification but highlight specific denominations—especially Cerina, Zitella, and Rosa—that warrant re-evaluation to improve the accuracy of varietal identification and conservation strategies.

## 5. Conclusions

The integration of regional and international genetic data is the first step toward a coordinated core European apple collection. This effort is essential to combat genetic erosion, as currently just four cultivars (Golden Delicious, Gala, Idared, and Red Delicious) account for 50% of commercial production in the EU. Local varieties from Abruzzo, supported by their molecular identity cards, represent an invaluable reservoir of genes for future breeding programs, offering potential solutions for climate change adaptation and resistance to emerging pathogens. In conclusion, this work provides useful tools for supporting the preservation of genetic diversity, ensuring the traceability of Abruzzo’s minor varieties and offering a scientific basis for their promotion. Nevertheless, further studies based on a larger and more balanced sampling are required to confirm and strengthen the conclusions regarding varietal diversity and genetic structure.

## Figures and Tables

**Table 1:** Dataset of samples collected from areas of the Abruzzo region.

Apple Variety	Sample ID
Cerina	CERFVTP1, CERFVTP2
Gelata	GELFVBP1, GELFVBP2, GELFVBP3, GELFVBP4, GELFVBP5, GELFMGP1, GELFVTP1, GELNCCP1, GELNCCP2, GELNCCP3, GELNXLP2, GELNXLP3
Limoncella	LIMFMGP1, LIMFMGP2, LIMFMGP3, LIMFMGP4, LIMFMGP5, LIMFVBP1, LIMFVBP2, LIMFVBP3, LIMFVTP1, LIMFVTP2, LIMFVTP3, LIMMQSP1, LIMMQSP2, LIMNCCP1, LIMNCCP2, LIMNXLP1, LIMNXLP2, LIMNXLP3
Renetta	RENFVBP1, RENFVBP2, RENFVBP3, RENFVTP1, RENFVTP2, RENFVTP3, RENNCCP1, RENNCCP2, RRUNXLP1, RRUNXLP2, RRUNXLP3, RRUNCCP1, RRUFVTP1, RRUFVTP2
Rosa	ROSFVTP1, ROSFVTP2, ROSMQSP1, ROSMQSP2, ROSMQSP4
Zitella	ZITNCCP1, ZITNXLP1, ZITNXLP2, ZITNXLP3, ZITMQSP1
Golden Delicious	GOLFVTP1

**Table 2:** SSR used for the characterization of apple accessions, including locus, number of alleles (NA) [reference], size range of the amplified fragment, primer sequence, fluorochrome, and multiplex (MP).

Locus	NA	Size Range (bp)	Forward Primer 5′→3′	Reverse Primer 5′→3′	Dye	MP
CH02d08	14 [8]	210–256	TCCAAAATGGCGTACCTCTC	GCAGACACTCACTCACTATCTCTC	Cy3	1
CH03d07	17 [10]	186–226	CAAATCAATGCAAAACTGTCA	GGCTTCTGGCCATGATTTTA	6Fam	1
CH03d12	25 [5]	96–157	GCCCAGAAGCAATAAGTAAACC	ATTGCTCCATGCATAAAGGG	Hex	1
CH05e03	15 [7]–26 [5]	154–198	CGAATATTTTCACTCTGACTGGG	CAAGTTGTTGTACTGCTCCGAC	Cy3	2
Hi05e07	26 [9]	213–266	CCCAAGTCCCTATCCCTCTC	GTTTATGGTGATGGTGTGAACGTG	6Fam	2
CH04c07	20 [9]	124–173	GGCCTTCCATGTCTCAGAAG	CCTCATGCCCTCCACTAACA	Hex	2
CH01f02	23 [9]–12 [6]	190–252	ACCACATTAGAGCAGTTGAGG	CTGGTTTGTTTTCCTCCAGC	Cy3	3
CH01a09	24 [9]	212–243	GATGTGGTTCCAGAAGCTAC	CACATGCATGAAAAGCATAT	6Fam	3
CH01g12	12 [8]–21 [5]	104–188	CCCACCAATCAAAAATCACC	TGAAGTATGGTGGTGCGTTC	Hex	3

**Table 3:** Genetic diversity parameters calculated for each locus: number of alleles (Na), effective number of alleles (Ne), percentage of rare alleles (Ra), observed heterozygosity (Ho), expected heterozygosity (He), inbreeding coefficient (F), and polymorphic information content (PIC). The standard deviation (Sd) quantifies the spread of observed values around the mean and serves as a measure of variability within the dataset.

Locus	Na	Ne	Ra	Ho	He	F	PIC
CH03d07	14	6.64	28.6	0.97	0.85	−0.142	0.833
CH03d12	15	6.68	20	0.81	0.85	0.052	0.835
CH02d08	10	5.91	10	0.78	0.83	0.066	0.809
Hi05e07	11	6.12	36.4	0.66	0.84	0.215	0.815
CH04c07	11	5.61	18.2	1	0.82	−0.217	0.798
CH05e03	12	5.75	25	0.82	0.83	0.006	0.807
CH01a09	15	7.45	6.7	0.97	0.87	−0.12	0.853
CH01g12	10	6.3	0	0.97	0.84	−0.153	0.822
CH01f02	12	6.85	16.7	0.7	0.85	0.179	0.837
Mean	12.22	6.37	17.96	0.85	0.84	−0.01	0.82
(Sd)	(3.76)	(1.82)	(10.99)	(0.26)	(0.23)	(0.14)	(0.23)

**Table 4:** List of rare alleles (frequency < 0.01) for each locus.

Locus	Allele (Accession)
CH03d07	163 (LIMONCELLA), 191 (ROSMQSP4), 215 (LIMMQSP1), 221 (RENETTARUGGINE)
CH03d12	102 (RENETTAVERDE), 129 (ZITMQSP1), 150 (RENETTAROSSA)
CH02d08	257 (RENETTARUGGINE)
Hi05e07	191 (ROSMQSP4), 192 (RENETTARUGGINE), 197 (RENETTAROSSA), 212 (ROSA)
CH04c07	116 (ROSMQSP4), 137 (LIMONCELLA)
CH05e03	160 (ROSMQSP4), 175 (RENETTAVERDE), 176 (ROSA)
CH01a09	184 (RENETTAROSSA)
CH01g12	-
CH01f02	196 (ROSA), 200 (RENETTARUGGINE)

**Table 5:** Groups of accessions with identical genetic profiles.

Group Name	Sample ID
LIM	LIMFMGP1, LIMFMGP2, LIMFMGP3, LIMFMGP4, LIMFMGP5, LIMFVBP1, LIMFVBP2, LIMFVTP1, LIMFVTP2, LIMFVTP3, LIMMQSP2, LIMNCCP1, LIMNCCP2, LIMNXLP1, LIMNXLP2, LIMNXLP3
LIM/ZIT	LIMFVBP3, ZITNXLP3
ZIT/CER	ZITELLA, CERINA, CERFVTP1, CERFVTP2, ZITNXLP1, RENFVBP1, GELFMGP1
ZIT	ZITNCCP1, ZITNXLP2
GEL	GELFVBP1, GELFVBP2, GELFVBP3, GELFVBP4, GELFVBP5, GELNCCP1, GELNCCP2, GELNCCP3, GELNXLP2, GELNXLP3
GOL	GOLDENCHIC, GOLDENEMLA, GOLDENHOLCOME, GOLFVTP1
REN1	RENFVBP2, RENFVBP3, RENFVTP1, RENFVTP2, RENFVTP3, RENNCCP1, RENNCCP2, RRUFVTP2
REN2	RRUNXLP1, RRUNXLP2, RRUNXLP3, RRUNCCP1
ROS	ROSFVTP1, ROSFVTP2

## Data Availability

The data presented in this study are contained within the article and the Supplementary Materials.

## Supplementary Materials

The following supporting information can be downloaded at: https://www.mdpi.com/article/10.3390/genes17070771/s1, Table S1: Genotypes of apples.
