# Supplementary material for: Genetic Analysis of Italian Local Apple Genotypes from the Abruzzo Region
Source: Genes (Basel). 2026 Jun 30;17(7):771. doi: 10.3390/genes17070771 (PMC13409560; doi:10.3390/genes17070771)
Supplement: Supplementary file 1 [file genes-17-00771-s001.zip › Table S1.pdf]

		CH03d07	CH03d12	CH02d08	Hi05e07	CH04c07	CH05e03	CH01a09	CH01g12	CH01f02
1	CERFVTP1	193-223	119	248	179-190-195	94-96	166-168	183-190	100-106	190
2	CERFVTP2	193-223	119	248	179-190-195	94-96	166-168	183-190	100-106	190
3	CERINA_OFA	193-223	119	248	179-190-195	94-96	166-168	183-190	100-106	190
7	GELFMGP1	193-223	119	248	179-190-195	94-96	166-168	183-190	100-106	190
8	GELFVBP1	193-199	119-149-151	213-248	179-190-195	94-96	166-168	190-197	106-129	173-190
9	GELFVBP2	193-199	119-149-151	213-248	179-190-195	94-96	166-168	190-197	106-129	173-190
10	GELFVBP3	193-199	119-149-151	213-248	179-190-195	94-96	166-168	190-197	106-129	173-190
11	GELFVBP4	193-199	119-149-151	213-248	179-190-195	94-96	166-168	190-197	106-129	173-190
12	GELFVBP5	193-199	119-149-151	213-248	179-190-195	94-96	166-168	190-197	106-129	173-190
13	GELFVTP1	189-193	119-149-151	230-248	179-190-195	94-120	167	183-196	100-135	163-190
14	GELNCCP1	193-199	119-149-151	213-248	179-190-195	94-96	166-168	190-197	106-129	173-190
15	GELNCCP2	193-199	119-149-151	213-248	179-190-195	94-96	166-168	190-197	106-129	173-190
16	GELNCCP3	193-199	119-149-151	213-248	179-190-195	94-96	166-168	190-197	106-129	173-190
17	GELNXLP2	193-199	119-149-151	213-248	179-190-195	94-96	166-168	190-197	106-129	173-190
18	GELNXLP3	193-199	119-149-151	213-248	179-190-195	94-96	166-168	190-197	106-129	173-190
19	GOLDENCHIC_OFA	186-203	119	225	210-226	94-112	181-187	201-203-205	102-144	173-182
20	GOLDENEMLA_OFA	186-203	119	225	210-226	94-112	181-187	201-203-205	102-144	173-182
21	GOLDENHOLCOME_OFA	186-203	119	225	210-226	94-112	181-187	201-203-205	102-144	173-182
22	GOLFVTP1	186-203	119	225	210-226	94-112	181-187	201-203-205	102-144	173-182
23	LIMFMGP1	204-223	123-131	226-230	182	96-112	158-172	201-207	100-108	179-197
24	LIMFMGP2	204-223	123-131	226-230	182	96-112	158-172	201-207	100-108	179-197
25	LIMFMGP3	204-223	123-131	226-230	182	96-112	158-172	201-207	100-108	179-197
26	LIMFMGP4	204-223	123-131	226-230	182	96-112	158-172	201-207	100-108	179-197
27	LIMFMGP5	204-223	123-131	226-230	182	96-112	158-172	201-207	100-108	179-197
28	LIMFVBP1	204-223	123-131	226-230	182	96-112	158-172	201-207	100-108	179-197
29	LIMFVBP2	204-223	123-131	226-230	182	96-112	158-172	201-207	100-108	179-197
30	LIMFVBP3	203-205	111	219-250	179-190-195	106-110	166	194-212	135-148	186-190
31	LIMFVTP1	204-223	123-131	226-230	182	96-112	158-172	201-207	100-108	179-197
32	LIMFVTP2	204-223	123-131	226-230	182	96-112	158-172	201-207	100-108	179-197
33	LIMFVTP3	204-223	123-131	226-230	182	96-112	158-172	201-207	100-108	179-197
34	LIMMQSP1	203-215	117-131	213-255	209-226	104-120	166	197-200	102	182-209
35	LIMMQSP2	204-223	123-131	226-230	182	96-112	158-172	201-207	100-108	179-197
36	LIMNCCP1	204-223	123-131	226-230	182	96-112	158-172	201-207	100-108	179-197
37	LIMNCCP2	204-223	123-131	226-230	182	96-112	158-172	201-207	100-108	179-197
38	LIMNXLP1	204-223	123-131	226-230	182	96-112	158-172	201-207	100-108	179-197
39	LIMNXLP2	204-223	123-131	226-230	182	96-112	158-172	201-207	100-108	179-197
40	LIMNXLP3	204-223	123-131	226-230	182	96-112	158-172	201-207	100-108	179-197
41	LIMONCELLA_OFA	163-223	104-119	226-255	179-190-210	110-137	166-170	183-207	106-135	173-182
42	RENETTAROSSA_OFA	189-223	117-150	226-230-255	179-197-210	106-110	170-181	184-193-207	104-106-148	182-186-209
43	RENETTARUGGINE_OFA	221	119-127	213-257	179-192-210	110-131	167	196-207	106-148	182-200
44	RENETTAVERDE_OFA	189-203-223	102-119	213-230-255	179-209-210	106-120	166-170-175	200-207	106-148	182-186
45	RENFVBP1	193-223	119	248	179-190-195	94-96	166-168	183-190	100-106	190
46	RENFVBP2	203-211-223	119-137	213-230-255	210-226	106-110	166-170	193-201-207	106-129-148	182
47	RENFVBP3	203-211-223	119-137	213-230-255	210-226	106-110	166-170	193-201-207	106-129-148	182
48	RENFVTP1	203-211-223	119-137	213-230-255	210-226	106-110	166-170	193-201-207	106-129-148	182
49	RENFVTP2	203-211-223	119-137	213-230-255	210-226	106-110	166-170	193-201-207	106-129-148	182
50	RENFVTP3	203-211-223	119-137	213-230-255	210-226	106-110	166-170	193-201-207	106-129-148	182
51	RENNCCP1	203-211-223	119-137	213-230-255	210-226	106-110	166-170	193-201-207	106-129-148	182
52	RENNCCP2	203-211-223	119-137	213-230-255	210-226	106-110	166-170	193-201-207	106-129-148	182
53	RRUNXLP1	203-211-223	119-137	213-230-255	210	106-110	166-170	193-201-207	106-129-148	182
54	RRUNXLP2	203-211-223	119-137	213-230-255	210	106-110	166-170	193-201-207	106-129-148	182
55	RRUNXLP3	203-211-223	119-137	213-230-255	210	106-110	166-170	193-201-207	106-129-148	182
56	RRUNCCP1	203-211-223	119-137	213-230-255	210	106-110	166-170	193-201-207	106-129-148	182
57	RRUFVTP2	203-211-223	119-137	213-230-255	210-226	106-110	166-170	193-201-207	106-129-148	182
64	RRUFVTP1	189-203	104-117-119	213-226	210-226	96-106-110	166-174	190-197	106-135	173-182
58	ROSA_OFA	189-199	104-131	213-219	179-195-212	106-110	174-176	196	102-129	196-209
59	ROSFVTP1	193-199	127-149-151	211-219	179-190	96-106	167	196-206-212	135-167	173-177-203
60	ROSFVTP2	193-199	127-149-151	211-219	179-190	96-106	167	196-206-212	135-167	173-177-203
61	ROSMQSP1	189-199	104-131	211-213	190	110-131	167	201	129-135	173
62	ROSMQSP2	199	127-149-151	211	190	96-106	167	196-212	135	173-177
63	ROSMQSP4	191-225	111-146-148	213-230	191	108-116	160-166	193-196	106-129	173-186
65	ZITELLA_OFA	193-223	119	248	179-190-195	94-96	166-168	183-190	100-106	190
66	ZITMQSP1	193-225	129-146-148	213	179-190	108-112	166	190-193	106-129	173-182
67	ZITNCCP1	199-203	119-149-151	255	179-190-210	104-110	166	198-200	100-104	163-209
68	ZITNXLP1	193-223	119	248	179-190-195	94-96	166-168	183-190	100-106	190
69	ZITNXLP2	199-203	119-149-151	255	179-190-210	104-110	166	198-200	100-104	163-209
70	ZITNXLP3	203-205	111	219-250	179-190-195	106-110	166	194-212	135-148	186-190
